# Reduced levels of intracellular calcium releasing in spermatozoa from asthenozoospermic patients

**DOI:** 10.1186/1477-7827-7-11

**Published:** 2009-02-06

**Authors:** Javier Espino, Matías Mediero, Graciela M Lozano, Ignacio Bejarano, Águeda Ortiz, Juan F García, José A Pariente, Ana B Rodríguez

**Affiliations:** 1 Department of Physiology, Faculty of Science, University of Extremadura, Badajoz, Spain; 2 Extremadura Center of Human Assisted Reproduction, Badajoz, Spain

## Abstract

**Background:**

Asthenozoospermia is one of the most common findings present in infertile males characterized by reduced or absent sperm motility, but its aetiology remains unknown in most cases. In addition, calcium is one of the most important ions regulating sperm motility. In this study we have investigated the progesterone-evoked intracellular calcium signal in ejaculated spermatozoa from men with normospermia or asthenozoospermia.

**Methods:**

Human ejaculates were obtained from healthy volunteers and asthenospermic men by masturbation after 4–5 days of abstinence. For determination of cytosolic free calcium concentration, spermatozoa were loaded with the fluorescent ratiometric calcium indicator Fura-2.

**Results:**

Treatment of spermatozoa from normospermic men with 20 micromolar progesterone plus 1 micromolar thapsigargin in a calcium free medium induced a typical transient increase in cytosolic free calcium concentration due to calcium release from internal stores. Similar results were obtained when spermatozoa were stimulated with progesterone alone. Subsequent addition of calcium to the external medium evoked a sustained elevation in cytosolic free calcium concentration indicative of capacitative calcium entry. However, when progesterone plus thapsigargin were administered to spermatozoa from patients with asthenozoospermia, calcium signal and subsequent calcium entry was much smaller compared to normospermic patients. As expected, pretreatment of normospermic spermatozoa with both the anti-progesterone receptor c262 antibody and with progesterone receptor antagonist RU-38486 decreased the calcium release induced by progesterone. Treatment of spermatozoa with cytochalasin D or jasplakinolide decreased the calcium entry evoked by depletion of internal calcium stores in normospermic patients, whereas these treatments proved to be ineffective at modifying the calcium entry in patients with asthenozoospermia.

**Conclusion:**

Our results suggest that spermatozoa from asthenozoospermic patients present a reduced responsiveness to progesterone.

## Background

It is well established that calcium signaling plays a pivotal role in sperm physiology, being intimately involved in the regulation of many aspects of mammalian sperm functions [1,2]. Control of motility, including hyperactivation and chemotaxis, is particularly dependent on intracellular free calcium concentration ([Ca^2+^]_i_) signaling in the principal piece of the flagellum and the midpiece [3-5]. In fact, abnormal motility might be explained by abnormally low cytoplasmic calcium [6,7]. Furthermore, it has been previously shown that capacitative calcium entry, via transient receptor potential (TRP) channels, may influence human sperm motility [8] and acrosome reaction [9].

Stimulation of human sperm with micromolar doses of progesterone increases [Ca^2+^]_i _in a biphasic manner [10,11], since the progesterone-activated signal comprises a transient [Ca^2+^]_i _'spike' (of 30–60 s duration at 37°C) followed by a sustained ramp or plateau. The mechanism by which progesterone elicits a response and subsequent events probably involves its interaction with a cell surface receptor on spermatozoa [12,13]. Therefore, the ability of progesterone to generate a response of [Ca^2+^]_i _in human spermatozoa has been directly correlated to fertilization success in vitro [14], indicating that this response is biologically important.

Progesterone is present in high (micromolar) concentrations in the follicular fluid [15,16] and is synthesized, both before and after ovulation, by the cells of the cumulus oophorus that surround the egg. Although the ability of progesterone to induce acrosome reaction in mammalian sperm is well established [17,18], it appears that progesterone-induced [Ca^2+^]_i _signaling might be involved on the regulation of flagellar activity, modulating motility and/or chemotaxis [19,20]. The finding that progesterone is a chemoattractant for human spermatozoa [21] indicates that at least one of the membrane progesterone receptors might act as a chemotaxis receptor [4].

Asthenospermia is a common cause in male infertility characterized by reduced forward motility (WHO grade a+b sperm motility <50% or a <25%) or absent sperm motility in fresh ejaculate, but its aetiology remains unknown in most cases. Any alteration in external and internal factors regulating sperm motion and in cellular structure and metabolisms involved in generating tail beat may result in defects in sperm motility and infertility [22]. In the last years, a significant decrease in the percentage of progesterone receptors has been found in men with asthenospermia [23]. In fact, different reports have suggested a relationship between male infertility and the inability of spermatozoa to respond to progesterone *in vitro *[24,25].

In this paper, we investigated the progesterone-evoked intracellular calcium signal and the role for the actin cytoskeleton in the store-mediated calcium entry in ejaculated spermatozoa from normospermic or asthenospermic men.

## Methods

### Chemicals

Progesterone, bovine serum albumin (BSA), RPMI-1640 medium, dimethyl BAPTA, RU-38486 and ethylene glycol-bis(2-aminoethylether)-N,N,N',N'-tetraacetic acid (EGTA) were from Sigma (Madrid, Spain). Fura-2 acetoxymethyl ester (fura-2/AM) and thapsigargin were from Invitrogen (Barcelona, Spain). Cytochalasin D and jasplakinolide were from Calbiochem (Darmstadt, Germany). Anti-progesterone receptor c262 mouse monoclonal antibody (PR c262) was obtained from Santa Cruz (Santa Cruz Biotechnology, Germany). All others reagents were of analytical grade.

### Spermatozoa preparation

Human semen was obtained from 37 healthy volunteers and 33 asthenozoospermic men at the Extremadura Center of Human Assisted Reproduction (Badajoz, Spain), as approved by local committees and in accordance with the Declaration of Helsinki. This study was approved by the institutional review board of the University of Extremadura and by the ethics committee of the Infantile Hospital (Badajoz, Spain). Each subject was ascertained to be in good health by means of their medical history and a clinical examination including routine laboratory tests and screening. The subjects all were nonsmokers, were not using any medication, and abstained from alcohol. Informed consent was obtained from all patients. Samples were collected by masturbation after 4 or 5 days of sexual abstinence and were allowed to liquefy at 37°C for 30 minutes. Semen was washed twice in RPMI medium (250 × g, 10 min), the supernatant was discarded, and the sperm pellet was resuspended in Na-HEPES solution containing the following (in mM): NaCl, 140; KCl, 4.7; CaCl_2_, 1.2; MgCl_2_, 1.1; glucose, 10; and HEPES, 10 (pH 7.4). The classical semen parameters of spermatozoa concentration, motility, and morphology were examined according to World Health Organization criteria [26]. Sperm concentration and motility were assessed by a computer assisted semen analysis (CASA) system. Our CASA system was based upon analysis of 25 consecutive, digitalized photographic images obtained from a single field at a 200 × magnification on dark field. The percentages of progressive motility were measured. The main criterion for classification of asthenozoospermic men was low sperm motility [27]. Normozoospermia was indicated by a sperm concentration of ≥ 20 × 10^6 ^cells/mL (mean ± SD = 62 ± 30 × 10^6 ^cells/mL), a progressive motility (grade a + b sperm motility) ≥ 50% (mean ± SD = 54.2 ± 4.1%) and a normal sperm morphology ≥ 14% (mean ± SD = 17 ± 3.6%). Asthenozoospermia was characterised by a sperm concentration of ≥ 20 × 10^6 ^cells/mL (mean ± SD = 42 ± 16 × 10^6 ^cells/mL) and a reduced forward motility (grade a+b sperm motility) <50% (mean ± SD = 23.3 ± 12.2%) or absent sperm motility, irrespective of the morphology results.

### Measurement of cytosolic free calcium concentration ([Ca^2+^]_c_)

Cells were loaded with fura-2 by incubation with 4 μM fura-2 acetoxymethyl ester (Fura-2 AM) for 30 minutes at room temperature, according to a procedure published elsewhere [28]. Once loaded, cells were washed and used within the next 2–4 hours. Fluorescence was recorded from 2 mL aliquots of magnetically stirred cellular suspension (2 × 10^8 ^cells/mL) at 37°C by using a Shimadzu spectrofluorophotometer with excitation wavelengths of 340 and 380 nm and emission at 505 nm. Changes in [Ca^2+^]_c _were monitored by using the fura-2 340/380 nm fluorescence ratio and were calibrated according to the method of Grynkiewicz et al. [29]. In experiments where calcium-free medium is indicated, calcium was omitted and ethylene glycol-bis(2-aminoethylether)-N,N,N',N'-tetraacetic acid (EGTA) was added.

Calcium entry and release were estimated using the integral of the rise in [Ca^2+^]_c _for 2.5 min after addition of CaCl_2 _or progesterone + thapsigargin, respectively [30]. Both calcium entry and release are expressed as nanomolar taking a sample every second (nM·s), as previously described [31].

### Statistical analysis

Data are expressed as means ± SD of the numbers of determinations. Analysis of statistical significance was performed by using the Student's *t*-test. P < 0.05 was considered to indicate a statistically significant difference.

## Results

### Asthenozoospermia and intracellular calcium mobilisation

**Figure 1:**
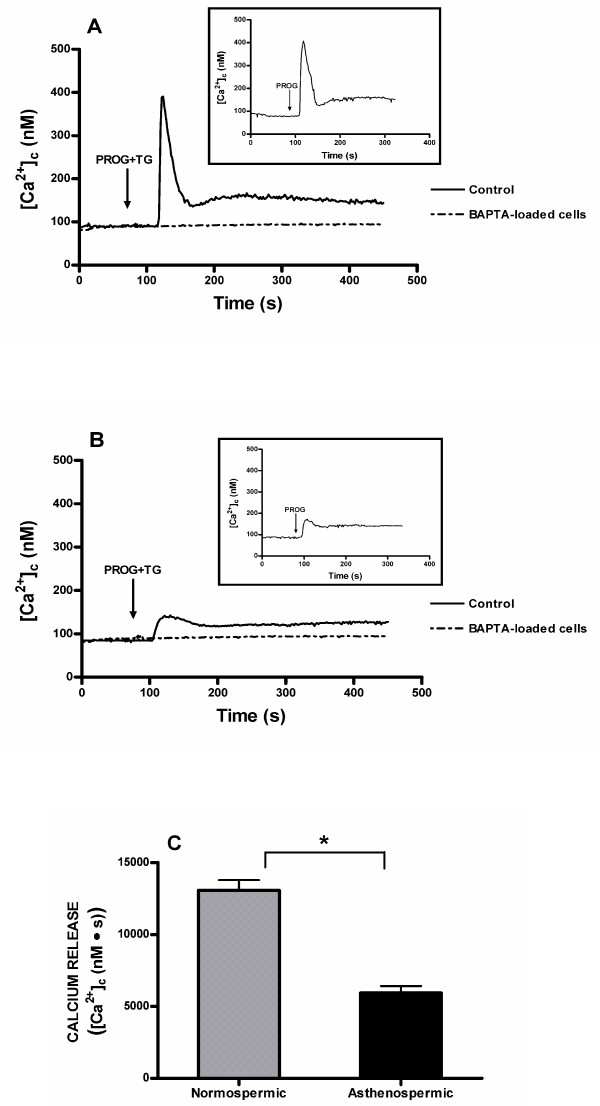
**Mobilization of calcium in response to progesterone in human spermatozoa from normospermic or asthenozoospermic patients**. Fura-2-loaded human spermatozoa from normospermic (A) or asthenozoospermic (B) patients were stimulated with 20 μM progesterone alone (PROG) (insets) or plus 1 μM thapsigargin (TG) in calcium-free solution (+ 1 mM EGTA), in the absence (control) or presence of dimethyl BAPTA (10 μM for 30 min). Traces are representative of five independent experiments. (C) Histogram represents the integral for 2.5 min of the calcium release, in normospermic and asthenozoospermic patients, calculated as described in Methods section. Values are means ± SD of five independent experiments. *P < 0.05.

In the absence of extracellular calcium (calcium-free medium), fura-2-loaded human spermatozoa were treated with 20 μM progesterone plus 1 μM thapsigargin. In spite of the fact that the presence of sarcoplasmic-endoplasmic reticulum calcium ATPase (SERCA) in sperm is still debated, we have used thapsigargin, a well-known SERCA inhibitor, to be sure that intracellular calcium stores were not refilled. In this regard, we have previously reported that human platelets possess two separate agonist-releasable calcium stores differentiated by the distinct sensitivity to thapsigargin [32,33]. As shown in Figure 1A, treatment with progesterone and thapsigargin induced a typical transient increase in [Ca^2+^]_c _due to calcium release from internal stores in spermatozoa from normospermic men. However, when progesterone plus thapsigargin were administered to spermatozoa from patients with asthenozoospermia, calcium signal was much smaller compared to calcium signal obtained in spermatozoa from normospermic men (Figure 1B). Similar results were obtained when spermatozoa were treated with progesterone alone (insets Fig. 1A and 1B) The integral of the rise in [Ca^2+^]_c _above basal for 2.5 min after addition of progesterone plus thapsigargin taking data every second were 13072.7 ± 697.1 and 5926.5 ± 475.3 nM·s in normospermic and asthenozoospermic men, respectively (Figure 1C; n = 7; P < 0.05). In addition, spermatozoa were loaded with dimethyl BAPTA, an intracellular calcium chelator, by incubating the cells for 30 minutes at 37°C with 10 μM dimethyl BAPTA-AM. As expected, dimethyl BAPTA loading prevented progesterone-evoked [Ca^2+^]_c _elevations in both normospermic (Figure 1A) and asthenozoospermic (Figure 1B) men.

Moreover, we evaluated the effect of 20 μM progesterone on progressive sperm motility measured by CASA system after 30 min of incubation. The treatment with progesterone caused a significant increase in the percentage of progressive motility in human spermatozoa from normospermic patients (54.2 ± 4.1 and 70.5 ± 2.3% in untreated and progesterone-treated spermatozoa, respectively), whereas progesterone was unable to modify the motility in spermatozoa from asthenozoospermic patients (23.3 ± 12.2 and 27.5 ± 10.3% in untreated and progesterone-treated spermatozoa, respectively).

**Figure 2:**
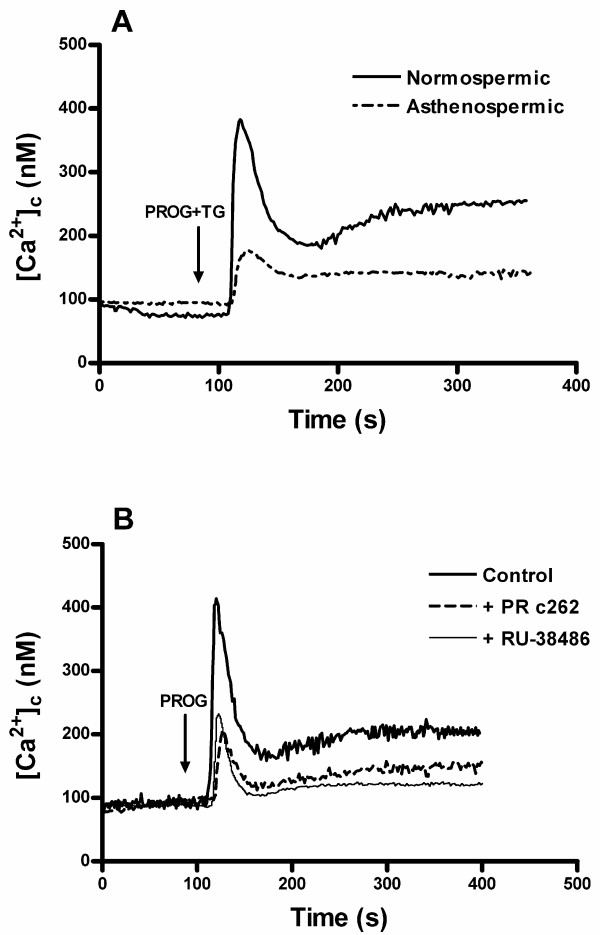
**Effect of the blockade of progesterone receptor on calcium mobilization evoked by progesterone in human spermatozoa**. (A) Fura-2-loaded human spermatozoa from normospermic and asthenozoospermic patients were stimulated with 20 μM progesterone (PROG) plus 1 μM thapsigargin (TG) in a calcium-normal solution (1.2 mM [Ca^2+^]_0_). (B) Fura-2-loaded human spermatozoa from normospermic patients were pretreated with the anti-progesterone receptor c262 antibody (PR c262) (1:10, final concentration 100 μg/ml for 30 min) or the progesterone receptor antagonist RU-38486 (50 μM for 30 min) and then stimulated with 20 μM progesterone (PROG) in a calcium-normal solution (1.2 mM [Ca^2+^]_0_). Traces are representative of 3–4 independent experiments.

Figure 2A demonstrates that the increase of [Ca^2+^]_c _induced by progesterone plus thapsigargin was also observed in the presence of extracellular calcium ([Ca^2+^]_0 _= 1.2 mM). In addition, we tested if progesterone receptor antibodies or antagonists would reverse the stimulatory effects of progesterone on calcium signal in normospermic spermatozoa. Preincubation of fura-2 loaded spermatozoa from normospermic patients with both the anti-progesterone receptor c262 antibody (PR c262) (1:10, final concentration 100 μg/ml) and the progesterone receptor antagonist RU-38486 (50 μM) for 30 min significantly reduced the progesterone-induced calcium release (Figure 2B). This clearly demonstrates that the blockade of progesterone receptors reduces the calcium mobilization induced by progesterone, and therefore normospermic spermatozoa behave as asthenozoospermic-like spermatozoa.

**Figure 3:**
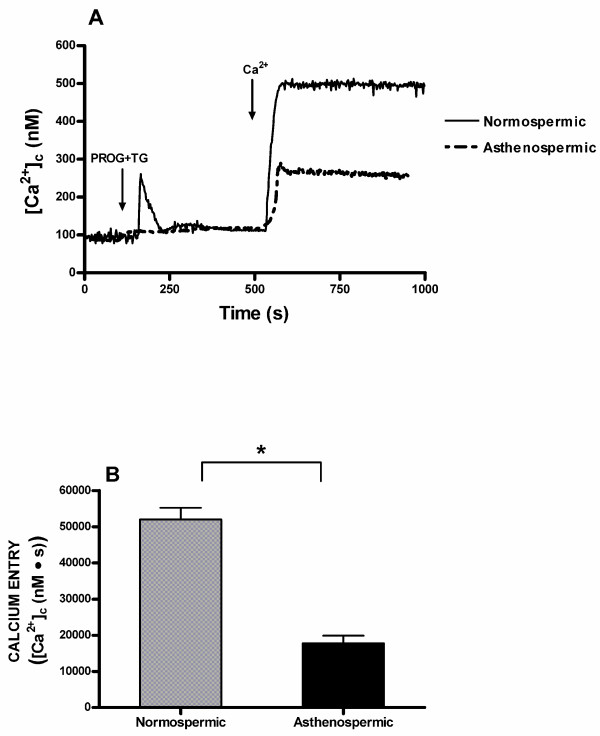
**Progesterone induced calcium entry in human spermatozoa from normospermic or asthenozoospermic patients**. (A) Fura-2-loaded human spermatozoa were treated with 20 μM progesterone (PROG) plus 1 μM thapsigargin (TG) for 6 min in a calcium-free medium (+ 100 μM EGTA) followed by addition of CaCl_2 _(300 μM) to initiate calcium entry. Traces are representative of seven independent experiments. (B) Histogram represents the integral for 2.5 min of the amount of calcium entry, in normospermic and asthenozoospermic patients, calculated as described in Methods section. Values are means ± SD of seven independent experiments. *P < 0.05.

Interestingly, subsequent addition of calcium (300 μM) to the suspension of progesterone plus thapsigargin-treated spermatozoa resulted in a detectable increase in [Ca^2+^]_c _indicative of calcium entry (Figure 3A). Similarly, subsequent calcium entry was significantly reduced (P < 0.05) in comparison to normospermic patients (Figure 3A). The integral of the rise in [Ca^2+^]_c _above basal for 2.5 min after addition of calcium taking data every second were 52003.2 ± 3219.4 and 17770.3 ± 2084.1 nM·s in normospermic and asthenozoospermic patients, respectively (Figure 3B; n = 7; P < 0.05).

### Effect of cytochalasin D and jasplakinolide on capacitative calcium entry in spermatozoa

**Figure 4:**
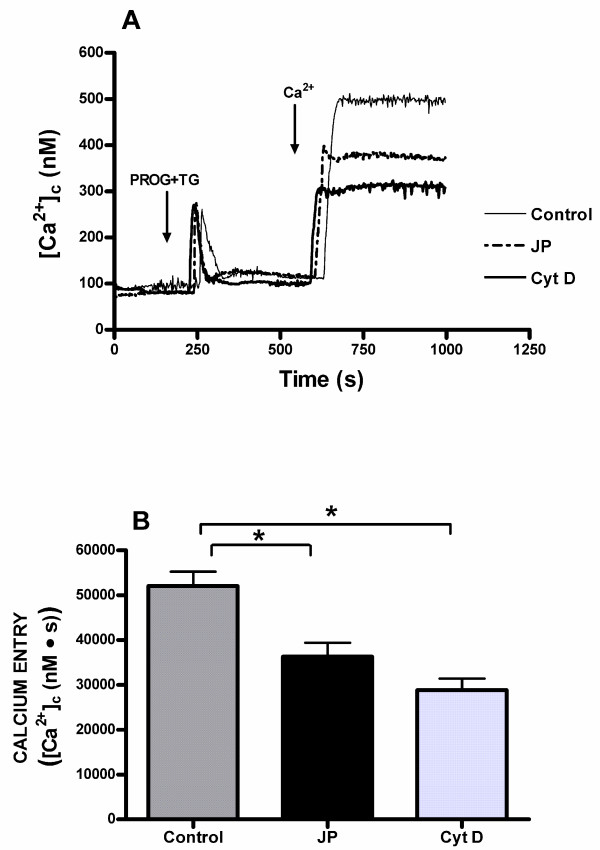
**Effects of cytochalasin D and jasplakinolide on progesterone induced calcium entry in human spermatozoa from normospermic patients**. (A) Fura-2-loaded human spermatozoa were preincubated at room temperature in the presence of 10 μM cytochalasin (Cyt D) for 40 min or 10 μM jasplakinolide (JP) for 30 min. Cells were then stimulated with 20 μM progesterone (PROG) plus 1 μM thapsigargin (TG) in calcium-free medium (+ 100 μM EGTA), and 6 min later CaCl_2 _(300 μM) was added to the medium to initiate calcium entry. Traces are representative of seven independent experiments. (B) Histogram represents the integral for 2.5 min of the amount of calcium entry, calculated as described in Methods section. Values are means ± SD of seven independent experiments. *P < 0.05.

Cytochalasin D, a widely utilized membrane-permeant inhibitor of actin polymerization which binds to the barbed end of actin filaments [34], and jasplakinolide, a cell-permeant peptide isolated from *Jaspis johnstoni *which induces polymerization and stabilization of actin filaments in vitro, but in vivo it can disrupt actin filaments and induce polymerization of monomeric actin into amorphous masses [35,36], are useful tools to further study the role of the actin cytoskeleton in store-mediated calcium entry. As shown in Figure 4A, pretreatment of human spermatozoa with both 10 μM cytochalasin D for 40 min and 10 μM jasplakinolide for 30 min at room temperature significantly diminished (p < 0.05) calcium entry evoked by depletion of internal calcium stores induced by progesterone plus thapsigargin in normospermic patients. The integral of the rise in [Ca^2+^]_c _above basal for 2.5 min after addition of calcium taking data every second were 28842.4 ± 2519.3 and 36256.1 ± 3129.7 nM·s in spermatozoa treated with cytochalasin D or jasplakinolide, respectively (Figure 4B; n = 7; P < 0.05).

**Figure 5:**
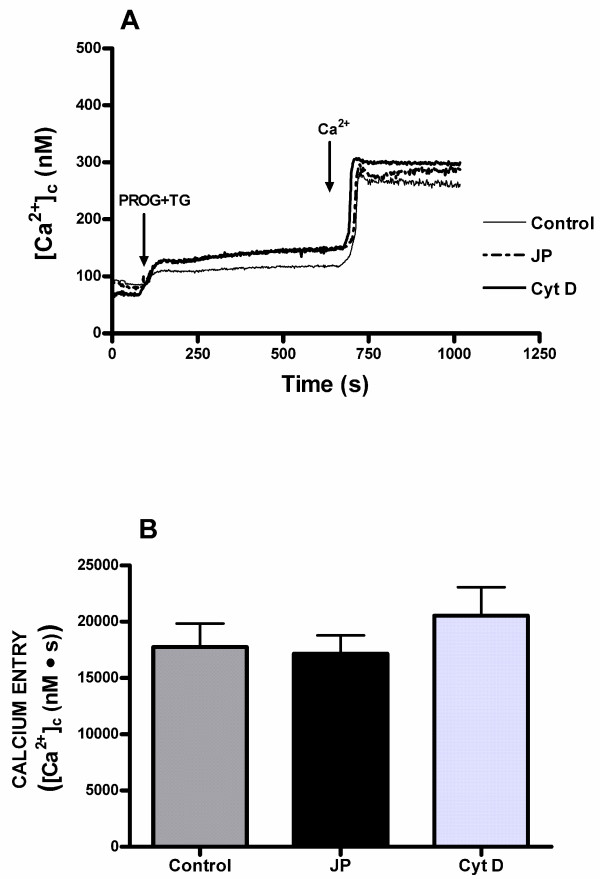
**Effects of cytochalasin D and jasplakinolide on progesterone induced calcium entry in human spermatozoa from asthenozoospermic patients**. (A) Fura-2-loaded human spermatozoa were preincubated at room temperature in the presence of 10 μM cytochalasin D (Cyt D) for 40 min or 10 μM jasplakinolide (JP) for 30 min. Cells were then stimulated with 20 μM progesterone (PROG) plus 1 μM thapsigargin (TG) in calcium-free medium (+ 100 μM EGTA), and 6 min later CaCl_2 _(300 μM) was added to the medium to initiate calcium entry. Traces are representative of seven independent experiments. (B) Histogram represents the integral for 2.5 min of the amount of calcium entry, in control, Cyt D-treated and JP-treated spermatozoa, calculated as described in Methods section. Values are means ± SD of seven independent experiments.

However, these treatments proved to be ineffective at modifying calcium entry in patients with asthenozoospermia (Figure 5A). The integral of the rise in [Ca^2+^]_c _above basal for 2.5 min after addition of calcium taking data every second were 20556.1 ± 2521.6 and 17175.3 ± 1624.9 nM·s in spermatozoa treated with cytochalasin D or jasplakinolide, respectively (Figure 5B; n = 7), which closely suggest that cytochalasin D and jasplakinolide are unable to affect the calcium entry evoked by depletion of intracellular calcium pools induced by progesterone plus thapsigargin in asthenozoospermic spermatozoa.

## Discussion

Progesterone, the most-studied and best-characterized calcium-mobilizing agonist of human sperm, caused a biphasic increase in [Ca^2+^]_c _from healthy donors as reported previously [18,28,37]. In addition, progesterone-induced [Ca^2+^]_c _transient showed very little sensitivity to the SERCA-inhibitor thapsigargin, since thapsigargin by itself had a negligible effect on calcium release from intracellular stores (Espino *et al*., unpublished observations). These findings are consistent with previous reports in human ejaculated spermatozoa [38,39] indicating that SERCAs do not contribute significantly to refill the progesterone-mobilized calcium store [37]. In addition, we cannot reject the involvement of secretory pathway calcium ATPase (SPCA), which is expressed in spermatozoa and mainly targeted to Golgi apparatus [40]. In fact, this non-SERCA store calcium-ATPase has been reported to be important in regulating [Ca^2+^]_i _[38].

Furthermore, our results have shown that capacitative calcium-influx occurs in sperm from normospermic men, which is consistent with a number of previous studies in sperm [8,11,39,41]. In the last years, capacitative calcium entry seems to be involved in the regulation of sperm motility, indicating that extracellular calcium plays a pivotal role in sperm motility [7,37].

In asthenozoospermic men, we have found that progesterone-induced calcium transient was undetectable and subsequent calcium entry was much smaller compared to normospermic patients. In addition, progesterone-induced calcium release in normospermic spermatozoa pretreated with both PR c262 and the progesterone receptor antagonist RU-38486 was similar to that obtained in spermatozoa from asthenozoospermic patients. This findings could be explained either by failure to localise a calcium signal to its site of action or by reduced or absent expression of progesterone receptors [23,42,43]. The reduced responsiveness to progesterone we found in sperm from asthenozoospermic subjects can be mainly due to decreased levels on membrane progesterone receptors, which could be translated in abnormal calcium signaling, and probably not to a direct effect on calcium release process. In fact, previous studies have reported a significant decrease in the percentage of progesterone receptors in asthenozoospermic men [23], and disturbance in the expression of membrane progesterone receptors might be involved in male infertility [44]. In addition, our results are in agreement with previous reports and suggest a strong relationship between calcium homeostasis, sperm motility, and male infertility. In fact, both reduced calcium/calmodulin (CaM) complex and intracellular calcium levels have been demonstrated in asthenozoospermic patients [45,46]. Moreover, different calcium channelopaties have been described for sperm calcium-permeable channels in asthenozoospermic patients [47,48].

On the other hand, both cytochalasin D, a widely used membrane-permeant inhibitor of actin polymerization, and jasplakinolide, a cell-permeant peptide which reorganizes actin filaments into a tight cortical layer adjacent to the plasma membrane [35,36], significantly reduced activation of store-mediated calcium entry in spermatozoa from normospermic men. These results suggest that vesicular trafficking might play an important role in store-operated calcium entry. Similar results have been previously obtained in both pancreatic acinar cells [49] and human platelets [50] when cells were stimulated with cholecystokinin or thrombin, respectively. These authors showed that disruption of actin cytoskeleton by cytochalasin D or stabilization of cortical actin barrier by jasplakinolide prevented the activation of store-mediated calcium entry, suggesting that actin cytoskeleton plays an important role in store-mediated calcium entry [49,50].

## Conclusion

Our results show that spermatozoa from asthenozoospermic patients present a reduced responsiveness to progesterone. We presume that disrupted calcium mobilization in spermatozoa from this group of patients might be associated with lower sperm motility and reduction of reproductive ability of these donors. Further studies are required to determine molecular mechanisms responsible for decreased progesterone-evoked intracellular calcium signal in spermatozoa from asthenozoospermic men.

## Abbreviations

[Ca^2+^]_c_: cytosolic free Ca^2+ ^concentration; [Ca^2+^]_i_: intracellular free Ca^2+ ^concentration; Cyt D: cytochalasin D; EGTA: ethylene glycol-bis(2-aminoethylether)-N,N,N',N'-tetraacetic acid; JP: jasplakinolide; PR c262: anti-progesterone receptor c262 antibody; TG: thapsigargin

## Competing interests

The authors declare that they have no competing interests.

## Authors' contributions

JE and MM carried out the experiments and wrote the manuscript. GML, AO and JFG collected sperm samples and carried out the analysis of sperm parameters. IB performed the statistical analysis and helped to write the manuscript. JAP and ABR conceived of the study, designed the experiments and discussed the results. All authors read and approved the final manuscript.
